# Oesophageal cancer incidence in the United States by race, sex, and histologic type, 1977–2005

**DOI:** 10.1038/sj.bjc.6605246

**Published:** 2009-08-11

**Authors:** M B Cook, W-H Chow, S S Devesa

**Affiliations:** 1 Division of Cancer Epidemiology and Genetics, Department of Health and Human Services, National Cancer Institute, National Institutes of Health, Bethesda, MD, USA

**Keywords:** adenocarcinoma, squamous cell, oesophagus, incidence, SEER program, trends

## Abstract

**Background:**

In the United States, the rates and temporal trends of oesophageal cancer overall and for the two predominant histologic types – adenocarcinoma (ADC) and squamous cell carcinoma (SCC) – differ between Blacks and Whites, but little is known with regard to the patterns among Asians/Pacific Islanders or Hispanics.

**Methods:**

Using the Surveillance, Epidemiology, and End Results programme data, we analysed oesophageal cancer incidence patterns by race, sex, and histologic type for the period 1977–2005.

**Results:**

Total oesophageal cancer incidence has been increasing among Whites only; the rates among all other race groups have declined. Moreover, rates among White men surpassed those among Blacks in 2004. Oesophageal SCC rates have been decreasing among virtually all racial/ethnic groups; rates among Hispanic and Asian/Pacific Islander men have been intermediate to those of Blacks and Whites, with rates among women being lower than those among Blacks or Whites. The ADC rates among Hispanic men may be rising, akin to the historical trends among Whites and Blacks. The sex ratios for these cancers also varied markedly.

**Conclusions:**

These observations may provide clues for aetiological research.

In 2002, there were an estimated 462 000 incident cases and 386 000 deaths attributable to oesophageal cancer worldwide, making this malignancy the eighth most common and sixth most deadly type of cancer (Parkin *et al*, 2005). It is primarily composed of two histologic types, squamous cell carcinoma (SCC) and adenocarcinoma (ADC), each apparently having a distinct aetiology (Holmes and Vaughan, 2007). A more in-depth analysis by race and sex may suggest avenues to elucidate their potential causal mechanisms.

Previous studies using the United States National Cancer Institute's Surveillance, Epidemiology, and End Results (SEER) cancer registry programme reported marked increases in oesophageal ADC, especially among White men, in contrast to decreases in SCC especially among Black men (Blot *et al*, 1991, 1993; Devesa *et al*, 1998; Brown and Devesa, 2002; Brown *et al*, 2008). Although several recent studies have used the SEER and the Centers for Disease Control and Prevention's National Program of Cancer Registries (NPCR) databases to expand these analyses, these have been based on shorter time periods and/or have focused on only certain racial/ethnic groups (Kubo and Corley, 2004; Wu *et al*, 2006, 2007; Mittal *et al*, 2008; Trivers *et al*, 2008). Therefore, we conducted a detailed descriptive analysis of SEER oesophageal cancer incidence patterns by race/ethnicity, sex, and histologic type for the years 1977/1992–2005.

## Materials and methods

The SEER cancer registry programme data (Surveillance Research Program, 2008) were used to prepare counts and incidence rates per 100 000 person-years (age-adjusted to the 2000 US standard population) of primary invasive oesophageal cancer (Fritz *et al*, 2000), topography codes C150 – 159), stratified by race, sex, and histology. Data for Whites and Blacks diagnosed during the period 1977–2005 were extracted from the November 2007 submission of the SEER 9 registries database (Surveillance Epidemiology and End Results (SEER) Program, 2007a). The expanded race variable is only available in the SEER 13 registries database (Surveillance Epidemiology and End Results (SEER) Program, 2007b). Thus, we extracted data for Whites (non-Hispanics only), Hispanics (Whites only), Blacks, American Indians/Alaskan Natives, and Asians/Pacific Islanders from the November 2007 submission of the SEER 13 registries database for cases diagnosed during the period 1992–2005. Counts and rates for American Indians/Alaskan Natives were restricted to the SEER 13 Contract Health Service Delivery Areas. The ICD-O-3 codes (2000) for the histology-specific analyses were SCC (8050–8084), ADC (8140–8575), and other and not specified (all remaining ICD-O-3 malignancy codes). For each race/ethnicity, male-to-female incidence rate ratios (MF IRRs) and 95% confidence intervals were calculated (Tiwari *et al*, 2006; Surveillance Research Program, 2008).

For graphs, rates for SEER 9 Whites and Blacks were calculated for six periods (1977–81, 1982–85, …, 2002–05). For all other races/ethnicities, data for SEER 13 for the three periods 1992–96, 1997–2001, and 2002–05 were calculated. Two or more successive data points with a minimum of 10 observations each were required for graphing, using OriginLab Corp (2007) with an aspect ratio of 40 years to one logarithmic cycle, such that a slope of 10 degrees portrays a change of 1% per year (Devesa *et al*, 1995).

## Results

During 1977–2005, more than 27 000 primary invasive oesophageal cancers were diagnosed among Whites (22 704) and Blacks (5003) in SEER 9 registries (Table 1). Rates among Black men and women were double (15.8 and 4.7 per 100 000 person-years, respectively) of those among White men and women (7.1 and 2.0, respectively). Rates among men were about triple of those among women. Squamous cell carcinoma accounted for 87% of all oesophageal cancer in Blacks but only for 45% in Whites. The SCC rate among Black men was four times that among White men (13.6 *vs* 2.7, respectively). Conversely, the male ADC rate among Whites was five times that among Blacks (3.7 *vs* 0.8, respectively). The Black/White racial patterns were similar in SEER 13 (1992–2005) and SEER 9 (1977–2005), although the SCC rates were lower and the ADC rates were higher in the latter amalgamated period.

During 1992–2005, Hispanics, compared with Whites, had lower total oesophagus, SCC, and ADC rates, except for SCC among men (2.6 *vs* 2.1). Despite the relatively small numbers, male rates among American Indians/Alaska Natives were intermediate compared with those of Whites and Blacks for total oesophageal cancer, SCC, and ADC; rates among female American Indians/Alaska Natives were similar to those of Whites, although based on much lower numbers of cases. Asians/Pacific Islanders had low overall and ADC rates among both men and women, and low SCC rates among women. The SCC rate among men, however, was higher than that among Whites and Hispanics, but still considerably lower than that among Blacks.

In the SEER 13 data (1992–2005), the MF IRR for total oesophageal cancer ranged from 2.9 among Blacks to around 4 for Whites, American Indians/Alaska Natives, and Asians/Pacific Islanders, and to 5.3 among Hispanics (Table 1). The MF IRRs for SCC were lower, ranging from 1.8 among Whites to 2.9 among Blacks and >4 among Hispanics, American Indians/Alaska Natives, and Asians/Pacific Islanders. In contrast, the MF IRRs for ADC were all >3 and exceeded 7 among both Whites and Hispanics.

**Figure 1:**
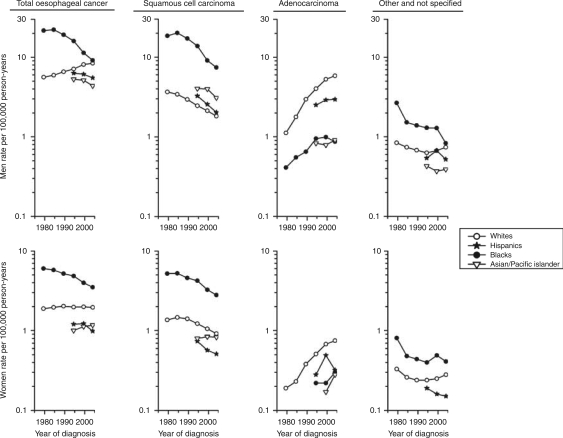
Oesophageal cancer incidence trends by histologic type, sex, and racial/ethnic group (SEER 9 and SEER 13). Rates are per 100 000 person-years, age-adjusted using US 2000 standard population data. ‘Whites’ refers to the rates for total Whites extracted from SEER 9. ‘Hispanics’ refers to the rates for Hispanics (Whites only) extracted from SEER 13.

Among men, total oesophageal cancer rates have been decreasing among Blacks since the mid-1980s and rising consistently among Whites, such that the Black–White IRR declined from 3.8 during the late 1970s to 1.1 during 2002–05 (Figure 1). In fact, the overall rates among Whites have been higher since 2004, with rates of 9.1 and 8.3 per 100 000 person-years in 2004–05 compared with 8.8 and 7.9 among Blacks (data not tabulated). Overall rates have also been declining among Hispanics and Asians/Pacific Islanders. Rates among Whites (non-Hispanics) were virtually identical to those among total Whites and those among American Indians/Alaska Natives were based on small numbers; thus, neither race/ethnicity is shown in the figure.

Among women, overall rates among Blacks have also been declining, although less rapidly than among men. Conversely, rates among White women have remained relatively stable, but among Hispanics and Asians/Pacific Islanders, rates were considerably lower and did not change significantly.

The rates of SCC have been declining for several decades among Blacks, Whites, and Hispanics of both sexes; only the rates among Asians/Pacific Islanders did not decrease notably. Among Asians/Pacific Islanders and Hispanics, they were consistently higher than those among Whites for men and lower for women. In contrast to the notable declines in SCC, ADC rates rose markedly, especially among Whites of both sexes and to a lesser extent among Blacks and Hispanic men.

Overall, cases with histologic type ‘Other and not specified’ accounted for 14% or less of all oesophageal cancer, and rates generally declined with time in most groups. It is unlikely that improved histologic specificity contributed meaningfully to the observed upward trends in ADC or dampened the decline in SCC.

## Discussion

Tobacco smoking and alcohol consumption are major known risk factors for oesophageal SCC in the United States (Freedman *et al*, 2007a). Historically, a higher proportion of Black men had a smoking habit relative to Whites (National Center for Health Statistics, 2007), and this may partly explain the racial disparity in SCC rates. Conversely, the proportion of Black and White women who smoke has not markedly differed, yet the rate of SCC among Black women was thrice that among White women. In addition, the proportion of US Asian men who smoke tobacco has been much lower than that of White men, although the SCC rate for Asian/Pacific Islander men was much higher. Alcohol consumption is much higher in Whites than in Blacks (National Center for Health Statistics, 2007), which is the reverse of their respective SCC incidence. Moreover, the proportion of Asian men who drink alcohol is equivalent to that of Blacks, yet Asian/Pacific Islander men rates are between those of White and Black men. These alcohol and tobacco exposures may indicate that other variables that mediate exposure dose (e.g., intensity, duration, passive smoking, and alcohol concentration) and racial/ethnic/sex differences in metabolic response may also contribute to the variation in SCC incidence. Conversely, exposure and outcome do not necessarily correlate, given the complexity of pathogenesis and masking by competing risk factors.

Additional SCC risk factors are body mass index (BMI) (Vaughan *et al*, 1995; Smith *et al*, 2008) and consumption of fruits and vegetables (Bosetti *et al*, 2000; Freedman *et al*, 2007b), both of which share an inverse relationship with this malignancy. Although a higher proportion of White men are overweight or obese, relative to Black men, the reverse is true for women (National Center for Health Statistics, 2007), which does not correspond to observed SCC incidence patterns. NHANES data suggest that between 1971 and 2002, fruit intake was similar for Whites and Blacks, whereas vegetable intake was slightly higher among Whites (Kant *et al*, 2007), a modest dissimilarity that is unlikely to explain the highly disparate SCC rates.

The major known risk factors for ADC are Barrett's oesophagus (Cook *et al*, 2007), gastro-oesophageal reflux disease (Lagergren *et al*, 1999), and overweight and obesity (Hampel *et al*, 2005). Recent data from two US studies (Corley *et al*, 2009; Wang *et al*, 2009) showed similar orders and relative incidences of Barrett's oesophagus among racial/ethnic groups to those of the most recent period of ADC incidence data presented in this study. The incidence of Barrett's oesophagus, meanwhile, seems to have been stable in the United States (Cameron *et al*, 1990; Macdonald *et al*, 1997; Conio *et al*, 2001; Corley *et al*, 2009), which is in marked contrast to ADC (Figure 1). It has been reported that the proportion presenting with reflux as an indication for endoscopy is highest in Whites, followed by Hispanics and then Blacks (Abrams *et al*, 2008), which is parallel to the incidence order of Barrett's oesophagus and ADC. In addition, evidence suggests that although Whites, Blacks, and Asians report a similar prevalence of heartburn, Whites are more likely to suffer from erosive reflux disease (Spechler *et al*, 2002; El-Serag *et al*, 2004; Corley *et al*, 2007). Finally, it seems that the prevalence of gastro-oesophageal reflux has been increasing in the United States (El-Serag and Sonnenberg, 1998). The totality of evidence supports the idea that racial/ethnic differences may originate in early pathogenesis.

Meta-analyses indicate that increasing BMI is associated with an increased risk of gastro-oesophageal reflux disease, Barrett's oesophagus, and ADC (Hampel *et al*, 2005; Cook *et al*, 2008; Smith *et al*, 2008; Kamat *et al*, 2009). The proportion of US adults classified as overweight or obese has been increasing rapidly since the mid-1970s (National Center for Health Statistics, 2007), which parallels that of ADC incidence. However, although White men have consistently had a higher proportion of overweight and obese individuals than Black men, the reverse is true for women, which does not accord with ADC incidence. Therefore, if BMI is a key determinant of ADC incidence, risk must be modified by other factors associated with race/ethnicity and/or sex, such as abdominal obesity (Corley *et al*, 2007).

Various indices of fruit and vegetable consumption consistently indicate an inverse relationship with ADC risk (Brown *et al*, 1995; Zhang *et al*, 1997; Cheng *et al*, 2000; Terry *et al*, 2001; Gonzalez *et al*, 2006). However, this exposure has not markedly varied between Whites and Blacks or over time (Kant *et al*, 2007). Tobacco smoking is positively associated with risk of ADC, but to a much lesser extent than SCC (Freedman *et al*, 2007a). Thus, differences in these exposures are less likely to be indicative of cancer incidence patterns, especially given the stronger risk factors of reflux and obesity.

The MF IRRs underline the fact that oesophageal cancer is a predominantly male disease with large variations by race/ethnicity and histology. The declines in SCC incidence, more rapid in men compared with women, are at least partly attributable to the decreased prevalence of tobacco smoking (Brown and Devesa, 2002; Cook *et al*, 2009). The sex ratio imbalance of ADC may originate during early pathogenesis (Cook *et al*, 2005), but causal exposures remain speculative (Cook *et al*, 2009).

Limitations of this analysis include the possibility of missing or misclassifying cases. However, SEER has extensive quality control procedures that have been in place for many years (Zippin *et al*, 1995; Nathan and Pawlik, 2008). Although data for the most recent years may have been under-reported (Clegg *et al*, 2007), delay adjustment of oesophageal cancer rates is smaller than that for many other cancers (Surveillance Epidemiology and End Results (SEER) Program, 2007c).

This analysis, the most comprehensive assessment of incidence patterns by race/ethnicity, sex, and histologic type to date, shows that oesophageal SCC rates have been decreasing among virtually all racial/ethnic groups, whereas ADC rates among Hispanic men may be rising, akin to the historical trends among Whites and Blacks.

## Conflict of interest

The authors declare no conflict of interest.

## Figures and Tables

**Table 1:** Oesophageal cancer count, incidence, and male-to-female incidence rate ratio with 95% confidence intervals by histologic type, sex, and racial/ethnic group (SEER 9 and SEER 13)

	**Men**			**Women**				
	**Count**	**Rate**	**95% CI**	**Count**	**Rate**	**95% CI**	**MF IRR**	**95% CI**
*All histologies*								
SEER 9 1977–2005								
White	16 574	7.1	(7.0–7.2)	6130	2.0	(1.9–2.0)	3.59	(3.48–3.70)
Black	3594	15.8	(15.3–16.3)	1409	4.7	(4.5–5.0)	3.34	(3.14–3.56)
SEER 13 1992–2005								
White	12 794	7.5	(7.4–7.6)	4370	1.9	(1.9–2.0)	3.85	(3.72–3.99)
Non-Hispanic (Whites)	11 712	7.7	(7.6–7.9)	4113	2.1	(2.0–2.1)	3.76	(3.63–3.90)
Hispanic	1082	6.0	(5.6–6.4)	257	1.1	(1.0–1.3)	5.28	(4.59–6.10)
Black	2015	11.5	(10.9–12.0)	934	3.9	(3.7–4.2)	2.91	(2.69–3.16)
American Indian/Alaska Native^a^	93	8.2	(6.5–10.1)	27	2.0	(1.3–2.9)	4.07	(2.62–6.59)
Asian/Pacific Islander	1024	4.9	(4.6–5.2)	284	1.1	(1.0–1.2)	4.45	(3.90–5.10)
								
*Squamous cell carcinoma*								
SEER 9 1977–2005								
White	6386	2.7	(2.6–2.8)	3825	1.2	(1.2–1.3)	2.18	(2.09–2.27)
Black	3138	13.6	(13.1–14.1)	1220	4.1	(3.8–4.3)	3.35	(3.13–3.59)
SEER 13 1992–2005								
White	3567	2.1	(2.0–2.2)	2389	1.1	(1.0–1.1)	1.95	(1.85–2.06)
Non-Hispanic (Whites)	3096	2.1	(2.0–2.1)	2249	1.1	(1.1–1.2)	1.80	(1.71–1.90)
Hispanic	471	2.6	(2.3–2.8)	140	0.6	(0.5–0.7)	4.30	(3.54–5.26)
Black	1675	9.4	(9.0–9.9)	780	3.3	(3.0–3.5)	2.89	(2.65–3.15)
American Indian/Alaska Native^a^	51	4.4	(3.2–5.8)	15	1.1	(0.6–1.8)	4.04	(2.23–7.85)
Asian/Pacific Islander	769	3.7	(3.4–4.0)	213	0.8	(0.7–0.9)	4.47	(3.83–5.23)
								
*Adenocarcinoma*								
SEER 9 1977–2005								
White	8614	3.7	(3.6–3.7)	1460	0.5	(0.4–0.5)	7.82	(7.39–8.27)
Black	169	0.8	(0.6–0.9)	52	0.2	(0.1–0.2)	4.30	(3.12–6.03)
SEER 13 1992–2005								
White	8158	4.8	(4.6–4.9)	1400	0.6	(0.6–0.7)	7.65	(7.22–8.10)
Non-Hispanic (Whites)	7643	5.0	(4.9–5.1)	1318	0.7	(0.6–0.7)	7.63	(7.19–8.11)
Hispanic	515	2.8	(2.6–3.1)	82	0.4	(0.3–0.5)	7.64	(6.02–9.84)
Black	175	1.0	(0.8–1.2)	64	0.3	(0.2–0.3)	3.64	(2.71–4.94)
American Indian/Alaska Native^a^	30	2.6	(1.7–3.8)	11	0.8	(0.4–1.5)	3.18	(1.55–7.17)
Asian/Pacific Islander	179	0.8	(0.7–1.0)	49	0.2	(0.1–0.3)	4.42	(3.20–6.21)
								
*Other and not specified*								
SEER 9 1977–2005								
White	1574	0.7	(0.7–0.7)	845	0.3	(0.2–0.3)	2.69	(2.47–2.93)
Black	287	1.4	(1.2–1.6)	137	0.5	(0.4–0.6)	2.87	(2.32–3.56)
SEER 13 1992–2005								
White	1069	0.7	(0.6–0.7)	581	0.2	(0.2–0.3)	2.63	(2.37–2.92)
Non-Hispanic (Whites)	973	0.7	(0.6–0.7)	546	0.3	(0.2–0.3)	2.57	(2.31–2.86)
Hispanic	96	0.6	(0.5–0.7)	35	0.2	(0.1–0.2)	3.58	(2.39–5.50)
Black	165	1.0	(0.9–1.2)	90	0.4	(0.3–0.5)	2.63	(2.01–3.46)
American Indian/Alaska Native^a^	12	1.2	(0.6–2.0)	1	∼	∼	∼	∼
Asian/Pacific Islander	76	0.4	(0.3–0.5)	22	0.1	(0.1–0.1)	4.39	(2.70–7.45)

MF IRR=male-to-female incidence rate ratio; CI=confidence interval.

a SEER-13 CHSDA (Contract Health Service Delivery Areas) counties.

Rates are per 100 000 person-years, age-adjusted using US 2000 standard population.

‘Whites’ refers to the rates for total Whites extracted from SEER 9. ‘Hispanics’ refers to the rates for Hispanics (Whites only) extracted from SEER 13. ∼, Rate and IRR not shown because there are fewer than 10 cases.

## References

[bib1] Abrams JA, Fields S, Lightdale CJ, Neugut AI (2008) Racial and ethnic disparities in the prevalence of Barrett's esophagus among patients who undergo upper endoscopy. Clin Gastroenterol Hepatol 6: 30–34 18063419 10.1016/j.cgh.2007.10.006 PMC3712273

[bib2] Blot WJ, Devesa SS, Kneller RW, Fraumeni Jr JF (1991) Rising incidence of adenocarcinoma of the esophagus and gastric cardia. JAMA 265: 1287–1289 1995976

[bib3] Blot WJ, Devesa SS, Fraumeni Jr JF (1993) Continuing climb in rates of esophageal adenocarcinoma: an update. JAMA 270: 1320 8360967

[bib4] Bosetti C, La Vecchia C, Talamini R, Simonato L, Zambon P, Negri E, Trichopoulos D, Lagiou P, Bardini R, Franceschi S (2000) Food groups and risk of squamous cell esophageal cancer in northern Italy. Int J Cancer 87: 289–294 10861489

[bib5] Brown LM, Devesa SS (2002) Epidemiologic trends in esophageal and gastric cancer in the United States. Surg Oncol Clin N Am 11: 235–256 12424848 10.1016/s1055-3207(02)00002-9

[bib6] Brown LM, Devesa SS, Chow WH (2008) Incidence of adenocarcinoma of the esophagus among white Americans by sex, stage, and age. J Natl Cancer Inst 100: 1184–1187 18695138 10.1093/jnci/djn211 PMC2518165

[bib7] Brown LM, Swanson CA, Gridley G, Swanson GM, Schoenberg JB, Greenberg RS, Silverman DT, Pottern LM, Hayes RB, Schwartz AG et al (1995) Adenocarcinoma of the esophagus: role of obesity and diet. J Natl Cancer Inst 87: 104–109 7707381 10.1093/jnci/87.2.104

[bib8] Cameron AJ, Zinsmeister AR, Ballard DJ, Carney JA (1990) Prevalence of columnar-lined (Barrett's) esophagus. Comparison of population-based clinical and autopsy findings. Gastroenterology 99: 918–922 2394347 10.1016/0016-5085(90)90607-3

[bib9] Cheng KK, Sharp L, McKinney PA, Logan RF, Chilvers CE, Cook-Mozaffari P, Ahmed A, Day NE (2000) A case-control study of oesophageal adenocarcinoma in women: a preventable disease. Br J Cancer 83: 127–132 10883680 10.1054/bjoc.2000.1121 PMC2374528

[bib10] Clegg LX, Reichman ME, Hankey BF, Miller BA, Lin YD, Johnson NJ, Schwartz SM, Bernstein L, Chen VW, Goodman MT, Gomez SL, Graff JJ, Lynch CF, Lin CC, Edwards BK (2007) Quality of race, Hispanic ethnicity, and immigrant status in population-based cancer registry data: implications for health disparity studies. Cancer Causes Control 18: 177–187 17219013 10.1007/s10552-006-0089-4

[bib11] Conio M, Cameron AJ, Romero Y, Branch CD, Schleck CD, Burgart LJ, Zinsmeister AR, Melton 3rd LJ, Locke 3rd GR (2001) Secular trends in the epidemiology and outcome of Barrett's oesophagus in Olmsted County, Minnesota. Gut 48: 304–309 11171817 10.1136/gut.48.3.304 PMC1760138

[bib12] Cook MB, Wild CP, Forman D (2005) A systematic review and meta-analysis of the sex ratio for Barrett's esophagus, erosive reflux disease, and nonerosive reflux disease. Am J Epidemiol 162: 1050–1061 16221805 10.1093/aje/kwi325

[bib13] Cook MB, Wild CP, Everett SM, Hardie LJ, Bani-Hani KE, Martin IG, Forman D (2007) Risk of mortality and cancer incidence in Barrett's esophagus. Cancer Epidemiol Biomarkers Prev 16: 2090–2096 17890521 10.1158/1055-9965.EPI-07-0432

[bib14] Cook MB, Greenwood DC, Hardie LJ, Wild CP, Forman D (2008) A systematic review and meta-analysis of the risk of increasing adiposity on Barrett's esophagus. Am J Gastroenterol 103: 292–300 17986313 10.1111/j.1572-0241.2007.01621.x

[bib15] Cook MB, Dawsey SM, Freedman ND, Inskip PD, Wichner SM, Quraishi SM, Devesa SS, McGlynn KA (2009) Sex disparities in cancer incidence by time period and age. Cancer Epidemiol Biomarkers Prev 18: 1174–1182 19293308 10.1158/1055-9965.EPI-08-1118 PMC2793271

[bib16] Corley DA, Kubo A, Zhao W (2007) Abdominal obesity, ethnicity and gastro-oesophageal reflux symptoms. Gut 56: 756–762 17047097 10.1136/gut.2006.109413 PMC1954862

[bib17] Corley DA, Kubo A, Levin TR, Block G, Habel L, Rumore G, Quesenberry C, Buffler P (2009) Race, ethnicity, sex and temporal differences in Barrett's oesophagus diagnosis: a large community-based study, 1994–2006. Gut 58: 182–188 18978173 10.1136/gut.2008.163360 PMC2671084

[bib18] Devesa SS, Donaldson J, Fears T (1995) Graphical presentation of trends in rates. Am J Epidemiol 141: 300–304 7840107 10.1093/aje/141.4.300

[bib19] Devesa SS, Blot WJ, Fraumeni Jr JF (1998) Changing patterns in the incidence of esophageal and gastric carcinoma in the United States. Cancer 83: 2049–2053 9827707

[bib20] El-Serag HB, Petersen NJ, Carter J, Graham DY, Richardson P, Genta RM, Rabeneck L (2004) Gastroesophageal reflux among different racial groups in the United States. Gastroenterology 126: 1692–1699 15188164 10.1053/j.gastro.2004.03.077

[bib21] El-Serag HB, Sonnenberg A (1998) Opposing time trends of peptic ulcer and reflux disease. Gut 43: 327–333 9863476 10.1136/gut.43.3.327 PMC1727258

[bib22] Freedman ND, Abnet CC, Leitzmann MF, Mouw T, Subar AF, Hollenbeck AR, Schatzkin A (2007a) A prospective study of tobacco, alcohol, and the risk of esophageal and gastric cancer subtypes. Am J Epidemiol 165: 1424–1433 17420181 10.1093/aje/kwm051

[bib23] Freedman ND, Park Y, Subar AF, Hollenbeck AR, Leitzmann MF, Schatzkin A, Abnet CC (2007b) Fruit and vegetable intake and esophageal cancer in a large prospective cohort study. Int J Cancer 121: 2753–2760 17691111 10.1002/ijc.22993

[bib24] Fritz AG, Percy C, Jack A, Shanmugaratnam K, Sobin L, Parkin DM, Whelan S (eds) (2000) International Classification of Diseases for Oncology. World Health Organization: Geneva

[bib25] Gonzalez CA, Pera G, Agudo A, Bueno-de-Mesquita HB, Ceroti M, Boeing H, Schulz M, Del Giudice G, Plebani M, Carneiro F, Berrino F, Sacerdote C, Tumino R, Panico S, Berglund G, Siman H, Hallmans G, Stenling R, Martinez C, Dorronsoro M, Barricarte A, Navarro C, Quiros JR, Allen N, Key TJ, Bingham S, Day NE, Linseisen J, Nagel G, Overvad K, Jensen MK, Olsen A, Tjonneland A, Buchner FL, Peeters PH, Numans ME, Clavel-Chapelon F, Boutron-Ruault MC, Roukos D, Trichopoulou A, Psaltopoulou T, Lund E, Casagrande C, Slimani N, Jenab M, Riboli E (2006) Fruit and vegetable intake and the risk of stomach and oesophagus adenocarcinoma in the European Prospective Investigation into Cancer and Nutrition (EPIC-EURGAST). Int J Cancer 118: 2559–2566 16380980 10.1002/ijc.21678

[bib26] Hampel H, Abraham NS, El-Serag HB (2005) Meta-analysis: obesity and the risk for gastroesophageal reflux disease and its complications. Ann Intern Med 143: 199–211 16061918 10.7326/0003-4819-143-3-200508020-00006

[bib27] Holmes RS, Vaughan TL (2007) Epidemiology and pathogenesis of esophageal cancer. Semin Radiat Oncol 17: 2–9 17185192 10.1016/j.semradonc.2006.09.003

[bib28] Kamat P, Wen S, Morris J, Anandasabapathy S (2009) Exploring the association between elevated body mass index and Barrett's esophagus: a systematic review and meta-analysis. Ann Thorac Surg 87: 655–662 19161814 10.1016/j.athoracsur.2008.08.003

[bib29] Kant AK, Graubard BI, Kumanyika SK (2007) Trends in black-white differentials in dietary intakes of USA adults, 1971–2002. Am J Prev Med 32: 264–272 17383557 10.1016/j.amepre.2006.12.011 PMC2001255

[bib30] Kubo A, Corley DA (2004) Marked multi-ethnic variation of esophageal and gastric cardia carcinomas within the United States. Am J Gastroenterol 99: 582–588 15089886 10.1111/j.1572-0241.2004.04131.x

[bib31] Lagergren J, Bergstrom R, Lindgren A, Nyren O (1999) Symptomatic gastroesophageal reflux as a risk factor for esophageal adenocarcinoma. N Engl J Med 340: 825–831 10080844 10.1056/NEJM199903183401101

[bib32] Macdonald CE, Wicks AC, Playford RJ (1997) Ten years' experience of screening patients with Barrett's oesophagus in a university teaching hospital. Gut 41: 303–307 9378382 10.1136/gut.41.3.303 PMC1891490

[bib33] Mittal A, Ona FV, Randolph ML, Yamamoto J (2008) A fresh look at esophageal cancer incidence among Asian/Pacific Islanders and Caucasians. Hawaii Med J 67: 206–208 18853891

[bib34] Nathan H, Pawlik TM (2008) Limitations of claims and registry data in surgical oncology research. Ann Surg Oncol 15: 415–423 17987343 10.1245/s10434-007-9658-3

[bib35] National Center for Health Statistics (2007) Health, United States, 2007. In With Chartbook on Trends in the Health of Americans. Hyattsville, MD 20698068

[bib36] OriginLab Corp (2007) Origin. Northampton, MA

[bib37] Parkin DM, Bray F, Ferlay J, Pisani P (2005) Global cancer statistics, 2002. CA Cancer J Clin 55: 74–108 15761078 10.3322/canjclin.55.2.74

[bib38] Smith M, Zhou M, Whitlock G, Yang G, Offer A, Hui G, Peto R, Huang Z, Chen Z (2008) Esophageal cancer and body mass index: results from a prospective study of 220,000 men in China and a meta-analysis of published studies. Int J Cancer 122: 1604–1610 18059032 10.1002/ijc.23198

[bib39] Spechler SJ, Jain SK, Tendler DA, Parker RA (2002) Racial differences in the frequency of symptoms and complications of gastro-oesophageal reflux disease. Aliment Pharmacol Ther 16: 1795–1800 12269973 10.1046/j.1365-2036.2002.01351.x

[bib40] Surveillance Epidemiology and End Results (SEER) Program (2007a) (www.seer.cancer.gov) SEER*Stat Database: Incidence - SEER 9 Regs Limited-Use, Nov 2007 Sub (1973–2005) <Katrina/Rita Population Adjustment> - Linked To County Attributes - Total U.S., 1969–2005 Counties, National Cancer Institute, DCCPS, Surveillance Research Program, Cancer Statistics Branch, released April 2008, based on the November 2007 submission

[bib41] Surveillance Epidemiology and End Results (SEER) Program (2007b) (www.seer.cancer.gov) SEER*Stat Database: Incidence - SEER 13 Regs Limited-Use, Nov 2007 Sub (1992–2005) <Katrina/Rita Population Adjustment> - Linked To County Attributes - Total U.S., 1969–2005 Counties, National Cancer Institute, DCCPS, Surveillance Research Program, Cancer Statistics Branch, released April 2008, based on the November 2007 submission

[bib42] Surveillance Epidemiology and End Results (SEER) Program (2007c) Cancer Statistics Review 1975–2005. In based on November 2007 SEER data submission, posted to the SEER web site, 2008, Ries LAG, Melbert D, Krapcho M, Stinchcomb DG, Howlader N, Horner MJ, Mariotto A, Miller BA, Feuer EJ, Altekruse SF, Lewis DR, Clegg L, Eisner MP, Reichman M, Edwards BK (eds). National Cancer Institute: Bethesda, MD

[bib43] Surveillance Research Program (2008) National Cancer Institute SEER*Stat software Vol. 2008

[bib44] Terry P, Lagergren J, Hansen H, Wolk A, Nyren O (2001) Fruit and vegetable consumption in the prevention of oesophageal and cardia cancers. Eur J Cancer Prev 10: 365–369 11535879 10.1097/00008469-200108000-00010

[bib45] Tiwari RC, Clegg LX, Zou Z (2006) Efficient interval estimation for age-adjusted cancer rates. Stat Methods Med Res 15: 547–569 17260923 10.1177/0962280206070621

[bib46] Trivers KF, Sabatino SA, Stewart SL (2008) Trends in esophageal cancer incidence by histology, United States, 1998-2003. Int J Cancer 123: 1422–1428 18546259 10.1002/ijc.23691

[bib47] Vaughan TL, Davis S, Kristal A, Thomas DB (1995) Obesity, alcohol, and tobacco as risk factors for cancers of the esophagus and gastric cardia: adenocarcinoma versus squamous cell carcinoma. Cancer Epidemiol Biomarkers Prev 4: 85–92 7742727

[bib48] Wang A, Mattek N, Holub J, Lieberman D, Eisen G (2009) Prevalence of Complicated Gastroesophageal Reflux Disease and Barrett's Esophagus Among Racial Groups in a Multi-Center Consortium. Dig Dis Sci 54: 964–971 19255852 10.1007/s10620-009-0742-3 PMC3856566

[bib49] Wu X, Chen VW, Ruiz B, Andrews P, Su LJ, Correa P (2006) Incidence of esophageal and gastric carcinomas among American Asians/Pacific Islanders, whites, and blacks: subsite and histology differences. Cancer 106: 683–692 16388522 10.1002/cncr.21542

[bib50] Wu X, Chen VW, Andrews PA, Ruiz B, Correa P (2007) Incidence of esophageal and gastric cancers among Hispanics, non-Hispanic Whites and non-Hispanic Blacks in the United States: subsite and histology differences. Cancer Causes Control 18: 585–593 17406989 10.1007/s10552-007-9000-1

[bib51] Zhang ZF, Kurtz RC, Yu GP, Sun M, Gargon N, Karpeh Jr M, Fein JS, Harlap S (1997) Adenocarcinomas of the esophagus and gastric cardia: the role of diet. Nutr Cancer 27: 298–309 9101561 10.1080/01635589709514541

[bib52] Zippin C, Lum D, Hankey BF (1995) Completeness of hospital cancer case reporting from the SEER Program of the National Cancer Institute. Cancer 76: 2343–2350 8635041 10.1002/1097-0142(19951201)76:11<2343::aid-cncr2820761124>3.0.co;2-#

